# The impact of diet and gut microbiota on development, treatment, and prognosis in prostate cancer

**DOI:** 10.3389/fnut.2025.1621389

**Published:** 2025-12-16

**Authors:** Guanmo Liu, Fan Yang, Wei Song, Rui Hou

**Affiliations:** 1 Department of General Surgery, Peking Union Medical College Hospital, Chinese Academy of Medical Sciences and Peking Union Medical College, Beijing, China; 2 Department of Urology, Peking Union Medical College Hospital, Chinese Academy of Medical Sciences and Peking Union Medical College, Beijing, China; 3 Institute of Clinical Medicine, National Infrastructures for Translational Medicine, Peking Union Medical College Hospital, Chinese Academy of Medical Sciences and Peking Union Medical College, Beijing, China

**Keywords:** prostate cancer, diet, nutrients, gut microbiota, diet-microbiota axis

## Abstract

Prostate cancer (PCa) progression is driven by a complex interplay of factors, including genetics, lifestyle, and environmental influences. Diet and gut microbiota have emerged as pivotal cancer development and treatment response modulators. This review delves into the intricate relationship between dietary modifications and gut microbiota, and their combined impact on PCa progression. Diets abundant in plant-based foods, fiber, and prebiotics promote beneficial gut microbiota profiles that support anti-inflammatory and anti-carcinogenic processes. In contrast, the Western dietary pattern, characterized by high levels of saturated fats and processed foods, may lead to dysbiosis, fostering pro-inflammatory conditions and the production of metabolites that enhance tumorigenesis. The gut microbiota influences the behavior of PCa through immune modulation, metabolic by-products, and interactions with systemic therapies. Emerging evidence, primarily derived from preclinical models or studies in non-PCa contexts, suggests that diet and gut microbiota may influence the development and progression of PCa. However, further PCa-specific clinical research is needed to validate these associations. Future research should prioritize the development of precise dietary recommendations and microbiota-targeted therapies that can be seamlessly incorporated into clinical practice for more personalized and effective cancer care.

## 1 Introduction

Prostate cancer (PCa) is the second most commonly diagnosed cancer and the fifth leading cause of cancer death among men worldwide, with more than 1,460,000 estimated cases and 396,000 deaths in 2022, with the highest incidence observed in Australia/New Zealand, North America, Northern Europe, and Latin America/Caribbean (1, 2). In contrast, mortality remains relatively high in Asia and Africa due to limited access to screening and treatment (2). In China, the incidence of prostate cancer has increased rapidly over the past decade, reflecting a transition toward Westernized dietary and lifestyle patterns (3). Understanding PCa risk requires examining genetic and environmental factors, highlighting the importance of considering genetic predispositions and lifestyle in cancer risk assessment (4, 5). Established risk factors include age, race, and family history. At the same time, modifiable influences such as diet, physical inactivity, obesity, and metabolic dysregulation have gained increasing recognition for their roles in tumor initiation and progression (6, 7).

In recent years, the gut microbiota’s involvement in PCa has become a focus of research attention. A comprehensive study employing 16S rRNA sequencing alongside animal models identified notable differences in the gut microbiota composition between castration-resistant PCa and castration-sensitive PCa mice, particularly emphasizing the phyla *Firmicutes* and *Bacteroidetes* (8). Due to technical limitations, the bioinformatics analysis in this study was restricted to genus-level resolution, which constitutes a limitation. Dysbiosis, featured by an imbalance in the gut microbial community, has been associated with various chronic diseases, including cancer (8). This dysbiosis may influence PCa progression through different metabolic pathways and specific microbial profiles may correlate with disease severity and treatment outcomes (9).

Despite growing interest, most evidence supporting diet–microbiota–PCa relationships derives from experimental models or observational studies, limiting causal inferences. Human prostate cancer–specific data remain insufficient, highlighting the need for translational studies to define mechanistic interactions and establish clinically applicable interventions. This review summarizes current evidence on how dietary patterns regulate gut microbiota and how these effects ultimately affect prostate cancer development, treatment response, and prognosis, while proposing future directions for evidence-based dietary and microbiota-targeted strategies.

## 2 Diet and lifestyle influence on PCa development

**Figure 1:**
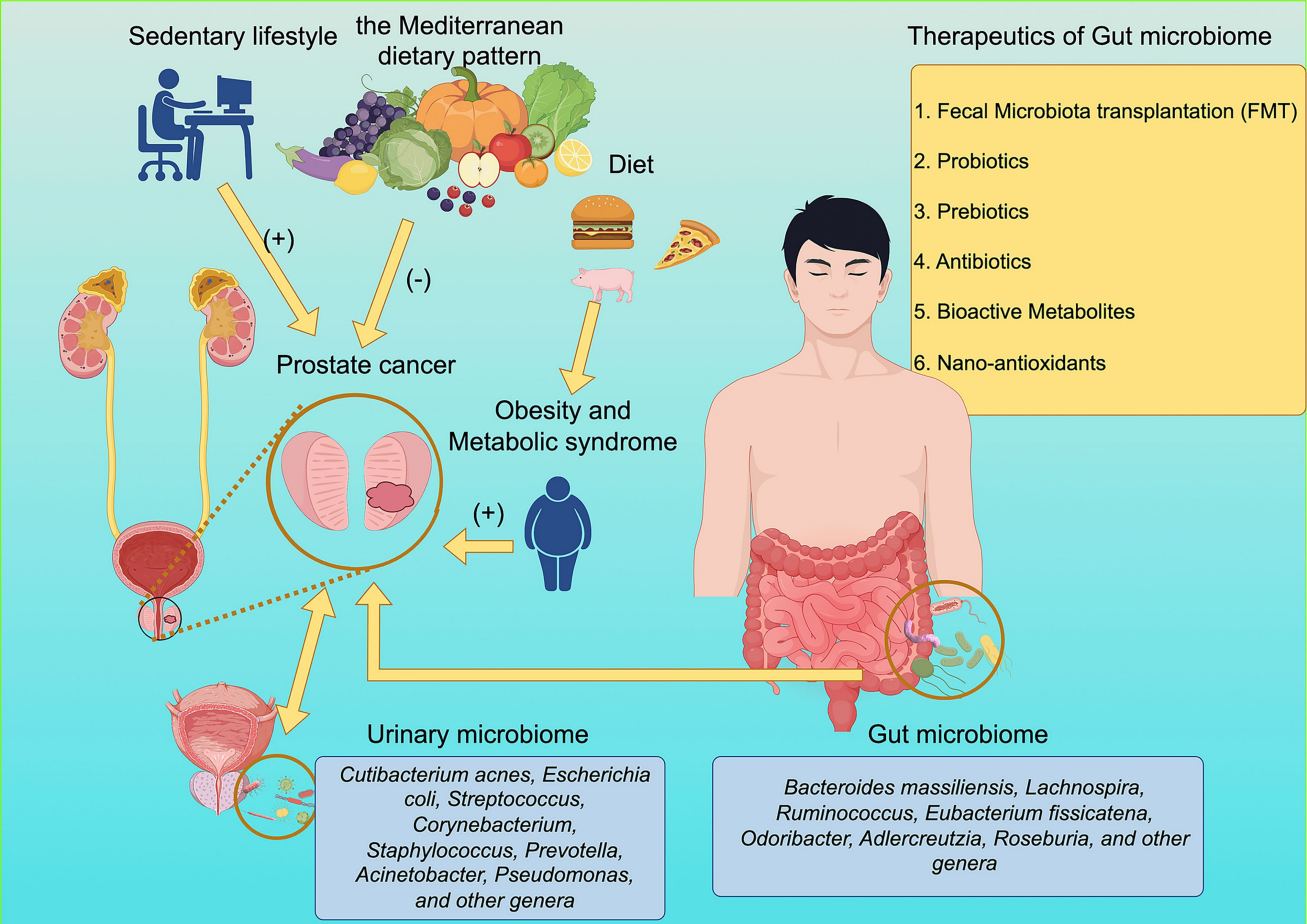
An integrated conceptual framework illustrating how lifestyle factors, dietary patterns, urinary microbiome, and gut microbiome influence PCa development. A sedentary lifestyle and Western dietary components (e.g., processed meat and fast food) exert potentially harmful effects on PCa, whereas the Mediterranean dietary pattern may confer protective effects. Western diet-associated metabolic disturbances contribute to obesity and metabolic syndrome, which in turn promote PCa progression. The urinary microbiome (including *Cutibacterium acnes*, *Escherichia coli*, *Streptococcus*, *Corynebacterium*, *Staphylococcus*, *Prevotella*, *Acinetobacter*, *Pseudomonas*, and other genera) may interact with both the prostate and bladder to modulate inflammatory or tumor-related pathways. The gut microbiome (including *Bacteroides massiliensis*, *Lachnospira*, *Ruminococcus*, *Eubacterium fissicatena*, *Odoribacter*, *Adlercreutzia*, *Roseburia*, and other genera) participates in metabolic regulation and may influence PCa directly or indirectly through obesity-related mechanisms. Potential therapeutic strategies targeting the gut microbiome include fecal microbiota transplantation, probiotics, prebiotics, antibiotics, bioactive metabolites, and nano-antioxidants. Source: Image generated using Figdraw.com.

Sedentary lifestyle was implicated as a risk factor for PCa detection and aggressiveness, substantiated by a study involving 2,408 men undergoing prostate biopsy (10). Men with the highest genetic risk were advised to have regular exercise rather than a sedentary lifestyle, which significantly reduced the risk of metastases and PCa-related mortality (11, 12). Concurrently, sedentary lifestyles have been associated with adverse metabolic and inflammatory profiles—such as insulin resistance and chronic low-grade inflammation—that may reduce immune surveillance and condition a tumor-promoting microenvironment (13–15) (Figure 1). Conversely, in men with PCa, greater vigorous physical activity has been linked to a less inflamed tumor immune landscape (16).

Epidemiological and observational studies have identified a correlation between unhealthy dietary habits, like high-fat diet and high-sucrose diet, and the increased risk of chronic non-communicable diseases, including various forms of cancer (17, 18). Dietary patterns also significantly influence PCa development and progression (19). Diets rich in red and processed meat – major feature of western dietary pattern, are consistently associated with a negative impact on gut microbiota diversity and functionality and a higher risk of PCa, particularly when meats are cooked at high temperatures, leading to the formation of carcinogenic compounds such as heterocyclic amines (HCAs) and polycyclic aromatic hydrocarbons (20). The California Collaborative Prostate Cancer Study found a strong correlation between advanced PCa risk and a large diet of red meat, particularly when cooked at high temperatures (21). The SABOR cohort study (*n* = 1,903) found that elevated dietary cholesterol and saturated fat intake were associated with increased PCa risk, probably by promoting androgen biosynthesis through altered lipid metabolism (22). Conversely, plant-based diets rich in fruits, vegetables, and whole grains—especially those aligned with the Mediterranean dietary pattern—are associated with reduced PCa risk (23–25). These diets provide antioxidants, polyphenols, and dietary fiber that exhibit anti-inflammatory and anti-cancer properties. Specific components, such as sulforaphane in cruciferous vegetables and phenolic compounds in olive oil, have demonstrated antiproliferative effects on PCa cells. Moreover, several studies in Mediterranean populations report a lower incidence of aggressive PCa among individuals adhering closely to this dietary pattern.

The Western dietary pattern induces gut dysbiosis and systemic inflammation, elevating ROS production and oxidative stress that collectively drive prostate tumorigenesis. In contrast, the antioxidant-rich Mediterranean diet counteracts these effects by reducing oxidative damage and promoting beneficial gut microbiota with anti-inflammatory properties (Figure 1). Emerging evidence highlights that specific dietary components influence PCa biology through well-defined oncogenic mechanisms. Diets high in red and processed meats are rich in heme iron, nitrates, and advanced glycation end products, which have been shown to increase oxidative stress and DNA damage in prostatic cells (26). Furthermore, saturated fats and cholesterol from Western diets may enhance intraprostatic androgen synthesis by upregulating key enzymes such as 17β-hydroxysteroid dehydrogenase and 5α-reductase, thus promoting PCa progression (27). Moreover, high-temperature cooking of meat leads to an increased formation of heterocyclic amines and advanced glycation end products (AGEs), which have attracted considerable attention due to their potential health implications (28). In contrast, polyphenols found in green tea and soy products have been associated with the inhibition of histone deacetylases and DNA methyltransferases, suggesting an epigenetic regulatory role (29). Cruciferous vegetables, through their sulforaphane content, may upregulate detoxifying enzymes and downregulate NF-κB signaling, reducing inflammation and oxidative stress in the prostate microenvironment (30). Collectively, these findings indicate that dietary components may influence PCa risk through multiple mechanisms, but the mechanistic insights remain limited and completely unclear, which requires further experimental or interventional studies.

Although current studies possess several limitations, such as food-frequency questionnaires used prone to recall bias, misclassification of nutrient intake and not considering the effect of marinades in the cooking of meats relative to the decreasing formation of HCAs (21, 22, 31). The association of diet and PCa, especially the tumorigenic effect of western diet has been widely acknowledged. Researches have been conducted to establish a clear relationship between specific nutrients and PCa.

## 3 Nutrient effects in reducing PCa risk

Several vitamins and minerals have been investigated for their protective roles in PCa (32, 33). Micronutrients, particularly antioxidants including vitamins A, C, D, E, and *β*-carotene, exhibit anti-inflammatory and anti-carcinogenic properties (34). Vitamin D regulates cell growth and differentiation; its antiproliferative effects in prostate cells involve interleukin-1α signaling and induction of cell-cycle arrest in progenitor/stem cells by 1,25-dihydroxyvitamin D₃ (35, 36). Vitamin E neutralizes free radicals and bolsters antioxidant defenses, potentially mitigating oxidative damage implicated in PCa pathogenesis (37). Selenium reduces oxidative stress biomarkers (e.g., urine 8-OHdG) and inflammation, with selenium-enriched yeast showing superior efficacy in PCa risk reduction compared to other forms (38, 39). Lipid intake significantly influences PCa development. Animal studies demonstrate that low-fat diets inhibit tumor growth and extend survival (40). Omega-3 fatty acids (present in walnuts, flaxseeds and fatty fish) lower PCa progression risk (41). However, the statistical power of this study was limited by the small sizes of key experimental cohorts, and the absence of independent replication warrants caution when extrapolating these results. A phase II randomized trial demonstrated that a low-fat diet supplemented with fish oil significantly altered the omega-6 to omega-3 ratio, decreased PCa cell proliferation and a lower omega-6 to omega-3 ratio in prostate tissue (42). Another study indicated a high omega-3, low omega-6 diet with fish oil for 1 year significantly reduced Ki-67 index (43). Ki-67, a nuclear proliferation marker, has been widely studied in multiple cancers. However, its clinical utility in PCa remains limited and controversial. Current guidelines do not recommend Ki-67 as a routine prognostic or treatment-guiding biomarker in PCa due to the lack of standardized detection protocols and cut-off values (44, 45).

Despite the accumulation of substantial observational data, the role of specific micronutrients in PCa is still not fully understood. The current study involved limitations in terms of baseline nutritional status, genetic polymorphisms, and study design. Moreover, interactions between micronutrients and hormone pathways, oxidative stress markers, and immune regulation remain important yet understudied. More fundamental research and stratified clinical trials are required to determine whether nutrient supplementation holds therapeutic value in PCa.

## 4 Association between gut microbiota and PCa

Apart from the direct influence of specific nutrients, different diet patterns may significantly influence the progression and treatment response of PCa through another route - gut microbiota. The gastrointestinal tract is home to a complex community of trillions of microorganisms known as the gut microbiota, which includes bacteria, viruses, fungi, and archaea. The gut microbiota can restrain tumor proliferation through anti-inflammatory responses and antioxidant effects. Conversely, specific microbial communities can promote tumor progression through ROS production and drug inactivation (46). The composition of the gut microbiota is highly diverse. It varies significantly between individuals and fluctuates over time due to diet, lifestyle, and health status (47). The gut microbiota not only aids in digestion and nutrient absorption but also plays a vital role in modulating the immune system and protecting against pathogens (48). Specifically, changes in the gut microbiota composition have been associated with elevated inflammatory markers, potentially contributing to the pathophysiology of PCa (8, 49, 50). Critically, comparative analyses revealed consistent alterations of gut microbial composition in patients with PCa. Several microbial taxa previously linked to pro-inflammatory responses — such as *Bacteroides massiliensis*, *Lachnospira*, *Ruminococcus*, *Eubacterium fissicatena*, and *Odoribacter* — were found to be relatively increased. In contrast, bacteria considered potentially beneficial, including *Adlercreutzia* and *Roseburia*, as well as short-chain fatty acid (SCFA)–producing groups like *Alphaproteobacteria*, showed a decreasing trend. Specifically, *Lachnospira* has been associated with high-risk PCa in human cohorts, whereas evidence for *Ruminococcus* pertains mainly to its enrichment in castration-resistant PCa compared with hormone-sensitive PCa (9, 51, 52). At present, certain members of the genus *Eubacterium* and *Clostridium* catalyzed the 7α-dehydroxylation of primary bile acids to deoxycholic and lithocholic acids, which could induce DNA damage in mammalian cells. However, this mechanism has not been validated for *Eubacterium fissicatena* or *Odoribacter* in PCa (53–57). In contrast, *Adlercreutzia* facilitates the conversion of dietary isoflavones into equol, exerting anti-androgenic and anti-proliferative effects (58), while *Roseburia* produces SCFAs such as butyrate, which inhibit histone deacetylases and suppress tumor growth (59). Furthermore, members of the genus *Bacteroides* may influence inflammatory pathways—for example, via lipopolysaccharides (LPS)-Toll-like receptor 4-NF-κB signaling described for other *Bacteroides*—but this mechanistic link remains to be validated for *Bacteroides massiliensis* in PCa (60–62). Although these microbial signatures underscore the potential of the microbiota as biomarkers for PCa, most current findings remains observational. The underlying mechanisms—such as how microbial metabolites modulate systemic inflammation or hormonal axes—warrant further elucidation through standardized metagenomic profiling and functional studies.

Recent studies suggest that gut microbiota can shape systemic and local immunity in PCa by modulating the balance of immune cell populations. Current evidence is primarily derived from animal models and gastrointestinal cancer cohorts; high-quality randomized controlled trials in PCa population cohorts are lacking. For instance, dysbiosis has been associated with increased infiltration of tumor-associated macrophages (TAMs) and myeloid-derived suppressor cells (MDSCs), which promote immunosuppression. SCFAs such as butyrate and propionate influence the differentiation of regulatory T cells (Tregs) and Th17 cells, potentially altering the Treg/Th17 ratio—a key immune checkpoint in cancer (63). Butyrate has also been shown to modulate cytokine expression, including IL-10 and TGF-*β*, contributing to an anti-inflammatory TME. Moreover, microbiota-derived metabolites may influence MHC-I expression on tumor cells and antigen-presenting cells, thereby affecting cytotoxic T-cell activation. Evidence also indicates that colonic dysbiosis may attenuate responses to immune checkpoint inhibitors by modulating interferon-*γ* signaling and PD-L1 expression (64). Although most of this research is preliminary and based on other tumor models, similar mechanisms may underlie PCa immune evasion and treatment resistance.

Emerging evidence has challenged the long-held assumption of a sterile urinary tract, highlighting the urinary microbiome (UMB) as a potential contributor to PCa initiation and progression. Several microbiome profiling studies have demonstrated altered microbial compositions in the urine or prostate tissue of PCa patients. Among them, Cutibacterium acnes has been frequently detected and is believed to promote carcinogenesis through chronic inflammation (65). Likewise, *Escherichia coli* (66), a major pathogen in bacterial prostatitis, may contribute to tumorigenesis by inducing recurrent epithelial damage and persistent inflammatory signaling. In addition, genera such as Streptococcus, Corynebacterium, Staphylococcus, Prevotella, Acinetobacter, and Pseudomonas show differential abundances between cancerous and noncancerous conditions, supporting the concept that microbial dysbiosis, rather than a single etiologic organism, may play a central role in shaping local tumor-promoting environments (67, 68). These microorganisms and their metabolites can modulate immune homeostasis, oxidative stress, and androgen metabolism, thereby remodeling the prostate tumor microenvironment. Although most current findings are associative and require mechanistic validation, the UMB represents a promising source of biomarkers and a potential mediator for precision prevention and treatment strategies in prostate cancer (Figure 1).

**Figure 2:**
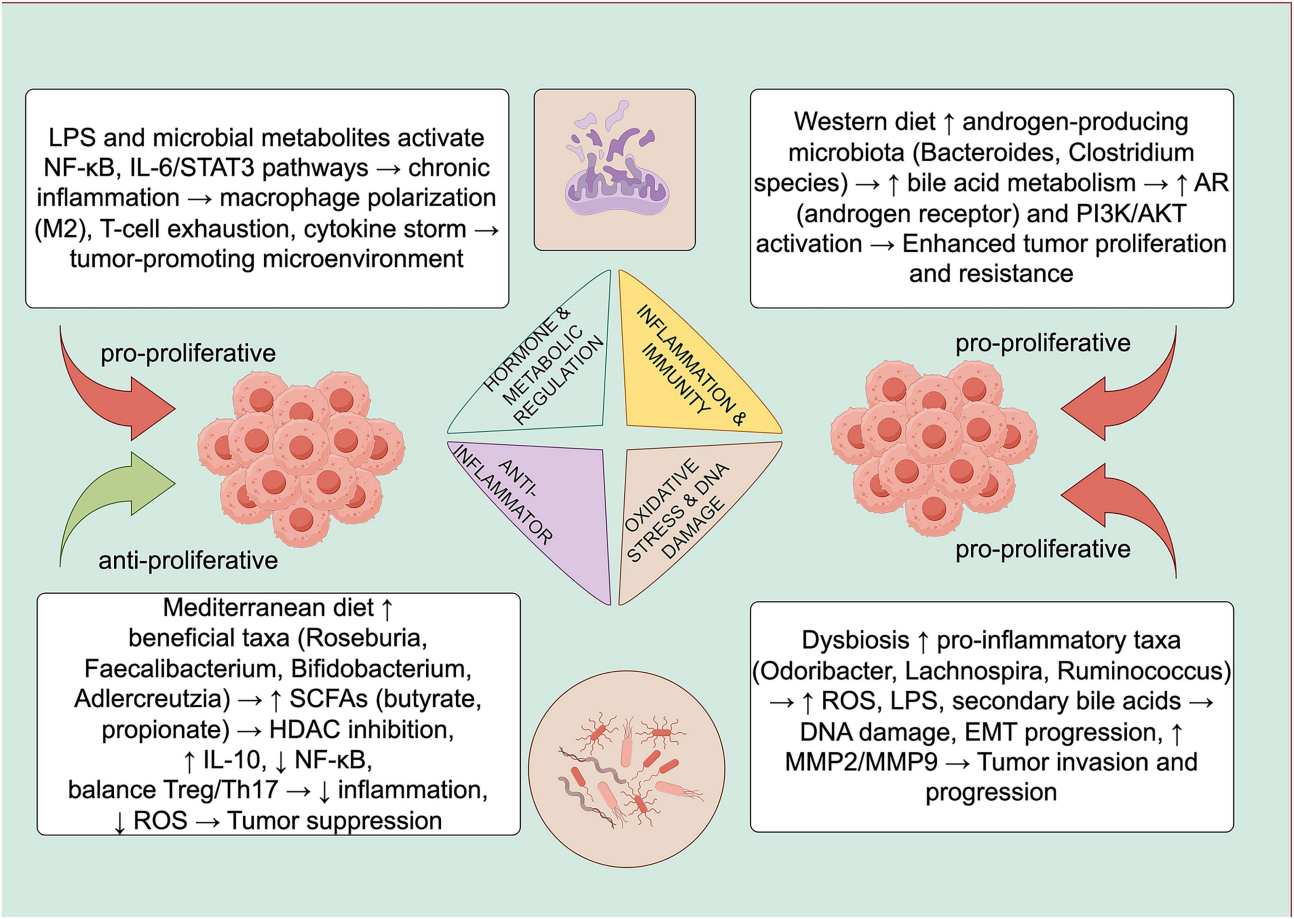
The gut microbiota exerts both tumor-suppressing and tumor-promoting effects through multiple mechanisms. Beneficial effects are mainly mediated by microbial metabolites such as SCFAs and polysaccharide A, leading to immune activation, anti-inflammatory molecule production, antioxidant activity, and drug activation. Tumor-promoting effects involve ROS generation, pro-inflammatory cytokine induction, oncogenic metabolite formation (e.g., secondary bile acids), and drug inactivation. Source: Image generated using Figdraw.com.

Collectively, beneficial microbiota-derived metabolites suppress tumor growth through anti-inflammatory signaling, antioxidant activity, and drug activation. In contrast, dysbiosis promotes prostate cancer by inducing ROS and pro-inflammatory cytokine production, generating oncogenic metabolites, and fostering therapy resistance via drug inactivation. Thus, the composition of the microbiota critically shapes the tumor microenvironment and its favorability for prostate cancer progression (Figure 2).

## 5 Diet-microbiota axis in PCa

Diet profoundly influences both the composition of gut microbiota and the progression of PCa, and recent studies highlight a functional axis linking dietary patterns, microbial metabolism, and PCa-related outcomes (Table 1). Characterized by high intake of red and processed meats, saturated fats, refined sugars, and low fiber, Western diets are associated with increased risk of PCa. This may be due to the formation of carcinogenic compounds (e.g., heterocyclic amines and polycyclic aromatic hydrocarbons) during high-temperature meat cooking, as well as excess dietary cholesterol promoting androgen biosynthesis, a key driver in PCa progression. In contrast, the Mediterranean dietary pattern, rich in olive oil, fruits, vegetables, legumes, whole grains, and moderate fish, has shown protective effects against Pca. Several population-based studies report that adherence to the Mediterranean diet correlates with reduced risk of aggressive disease (Gleason >6 or advanced clinical stage) and lower PCa-specific mortality (69). The mechanistic explanation involves dietary modulation of the gut microbiota and its metabolic byproducts. Diets high in fiber, polyphenols, and plant-based components support the growth of beneficial bacteria such as *Bifidobacterium* and *Faecalibacterium*, which ferment dietary fiber into SCFAs such as butyrate, propionate, and acetate (70). These SCFAs play pivotal roles in maintaining intestinal barrier function, reducing systemic inflammation, and regulating immune surveillance. Butyrate, in particular, has been shown to inhibit histone deacetylases (HDACs), alter gene expression, and suppress tumor growth in prostate cell models.

**Table 1:** Comparison of dietary patterns, gut microbiota changes, and PCa–related impacts.

Dietary pattern	Key features	Gut microbiota changes	Potential impacts on PCa	References
Western diet	High in red/processed meat, sugar, saturated fats	↓ SCFA-producing bacteria, ↑ *Bacteroides*, ↑ ROS-producing species	↑ Inflammation, ↑ androgen biosynthesis via cholesterol metabolism, ↑ PCa progression	(23, 29)
Mediterranean diet	Rich in fruits, vegetables, legumes, olive oil, moderate fish	↑ *Bifidobacterium*, *Lactobacillus*, ↑ SCFA production (butyrate, acetate)	↓ Oxidative stress, ↑ anti-inflammatory effects, ↓ PCa incidence and progression	(48)
Plant-based diet	High fiber, antioxidants, phytochemicals	↑ *Prevotella*, ↑ microbial diversity, ↑ SCFA production	↓ ROS, ↓ DNA damage, improved immune regulation, ↓PCa incidence	(20–22)
High-fat/High-sucrose Diet	Rich in cholesterol, processed carbs	↓ microbial diversity, ↑ *Firmicutes/Bacteroidetes* ratio	↑ Metabolic inflammation, ↑ PCa progression via lipid- and hormone-mediated pathways	(19, 23)
Probiotic/Prebiotic use	Supplementation with beneficial bacteria	↑ SCFA-producing strains, ↓ pathogenic bacteria	↓ Serum urea, ↑ gut barrier integrity, potential for better immune response and post-treatment recovery in PCa	(75, 80)

Conversely, Western dietary patterns reduce microbial diversity and increase the abundance of pro-inflammatory taxa (71). This microbial dysbiosis contributes to oxidative stress, chronic inflammation, and immune evasion in the TME. For example, elevated levels of *Bacteroides* and *Alistipes*, often observed in high-fat diets, are associated with the production of LPS and ROS that may promote tumorigenesis. Preclinical models have demonstrated that SCFA-producing bacteria can modulate Treg/Th17 balance (72), but such findings remain largely derived from murine or *in vitro* studies.

The gut microbiota’s influence extends beyond PCa pathogenesis to surgical outcomes, where optimal recovery relies on microbial homeostasis to regulate immune function and control inflammation. Surgical procedures may disrupt this balance, leading to complications, but probiotics and symbiotics administration has been shown to reduce infection risks such as surgical site infections and mitigate inflammation by preserving intestinal barrier integrity and improving motility (73, 74). These interventions can shorten hospital stays and alleviate postoperative pain (75). Notably, gut microbial dysbiosis may exacerbate immune dysfunction, particularly in older patients, potentially worsening postoperative inflammation and delaying recovery (76, 77). In PCa patients undergoing radical prostatectomy, the study suggested that gut and urinary microbiota composition might influence surgical recovery and complication rates (75), highlighting opportunities for preoperative microbiota assessment to guide personalized care strategies (78). A comprehensive understanding of diet-microbiota interactions could thus inform both preventive approaches through dietary interventions and perioperative optimization via probiotic supplementation to enhance immune function and clinical outcomes (79).

Taken together, the diet-microbiota axis represents a modifiable target across the PCa continuum from primary prevention to postoperative management. Future clinical studies should integrate metagenomic sequencing and metabolomic profiling to evaluate how diet-driven microbial modulation impacts PCa progression and surgical recovery, bridging mechanistic insights with translational applications.

## 6 Gut microbiota-targeted interventions

Microbiota-targeted interventions, such as probiotics, prebiotics, and fecal microbiota transplantation (FMT), are gaining attention for their potential to improve perioperative outcomes by restoring gut balance, reducing inflammation, and promoting recovery. Further research is needed to optimize these interventions and establish standardized protocols for clinical use (80, 81).

Prebiotics (such as inulin and fructooligosaccharides) and probiotics (e.g., *Lactobacillus rhamnosus* GG, *Bifidobacterium longum*) have shown benefits in other malignancies by improving gut health, lowering postoperative infection rates, reducing serum urea levels in PCa patients with comorbid chronic kidney disease, and promoting faster return to normal gastrointestinal function (82). Prebiotics, such as inulin and fructo-oligosaccharides, are crucial to restore a normal gut microbiota and are particularly helpful in PCa patients with perioperative use of antibiotics (83). The gut microbiota-brain axis was also important as alterations in gut microbiota could influence pain perception and psychological well-being, which were critical for cancer patients during recovery (84). Furthermore, the administration of antibiotics, although necessary for preventing perioperative infections, leads to significant dysbiosis in the gut microbiota (85). This disruption can adversely affect gut health, immune function, and overall recovery.

FMT probably accelerated recovery and overall health outcomes in patients undergoing surgical procedures for malignancies, such as PCa. The supposed mechanism for this effect involves the restoration of gut microbiota diversity, which is frequently compromised by both the malignancy and cancer-related treatments, including antibiotic use and chemotherapy. By reestablishing a healthy microbiota, FMT may help mitigate complications, decrease the risk of infections, and improve surgical outcomes for PCa patients (8, 9). This novel application of FMT indicates that it could serve as a valuable adjunctive therapy in the management of PCa patients, particularly those undergoing major surgical interventions (86–89). However, the use of FMT in immunocompromised individuals necessitates careful consideration due to potential risks, including infections and systemic inflammatory responses. A meta-analysis published in 2025 concluded that FMT had comparable effectiveness and safety in immunocompromised and immunocompetent patients (90). Nevertheless, the American Gastroenterological Association guidelines suggested against the use of FMT–based therapies in severely immunocompromised adults, citing insufficient evidence regarding safety (91). Similarly, high-dose probiotics have been associated with a reduced risk of infections in certain immunocompromised populations. There have been reports of bloodstream infections associated with probiotic use in immunocompromised cancer patients, particularly with strains such as *Lactobacillus* (92). Therefore, their use in immunocompromised individuals requires careful consideration of the associated risks. Adherence to safety protocols, individualized patient assessment, and vigilant monitoring are essential to mitigate potential adverse outcomes.

Despite encouraging data from gastrointestinal malignancies, no large-scale clinical trials have yet assessed the direct efficacy of microbiota-modulating therapies in PCa populations. Anatomical and immunological differences between cancer types complicate direct extrapolation. Therefore, well-designed PCa-specific trials are needed. These could include prospective microbiome profiling in patients under active surveillance or androgen deprivation therapy (ADT), as well as pilot intervention trials using SCFA-enhancing prebiotics and probiotics. Such studies would help identify microbial signatures associated with disease aggressiveness and inform personalized interventions.

From a translational perspective, microbiota modulation may also facilitate PCa immunotherapy by reprogramming immune phenotypes in the tumor microenvironment (TME). Preclinical data suggest that certain *Bifidobacterium* strains enhance dendritic cell maturation and augment CD8 + T cell antitumor responses (93). While these findings are mainly derived from melanoma or colorectal cancer models, they highlight a promising avenue for adjuvant strategies in PCa. Future research should incorporate immune phenotyping, including Treg/Th17 ratios, cytokine profiling, and MHC expression analyses, to explore how microbiome-directed interventions could enhance immunotherapeutic efficacy.

## 7 Future perspectives

Personalized nutrition scheme is gaining traction as a key strategy in adjusting the gut microbiota to improve prognosis outcomes of PCa. Understanding individual variations in gut microbiota composition could pave the way for tailored dietary recommendations to enhance treatment responses. For PCa patients, personalized diets that include specific prebiotics, probiotics, and nutrient-rich foods may help optimize gut health and strengthen treatment efficacy.

In the future, clinicians can leverage advances in metagenomic sequencing and metabolomics to identify predictive biomarkers of treatment response, to develop tailored nutritional plans that promote beneficial microbial compositions. Mechanism studies are needed to elucidate how specific microbial metabolites directly regulate androgen signaling, immune evasion, or oxidative stress pathways in PCa. Additionally, rigorous randomized controlled trials are essential to evaluate the long-term efficacy of dietary interventions such as the Mediterranean dietary pattern and omega-3 supplementation, combined with microbiota-targeted therapies (probiotics, prebiotics, or FMT) in improving oncologic outcomes, reducing treatment toxicity, and mitigating recurrence. Finally, longitudinal studies in diverse populations, particularly in regions with rising PCa incidence like China, will address ethnic and environmental variability, ensuring globally applicable strategies to harness diet-microbiota synergies for PCa management.

Despite growing interest in the gut–prostate axis, several confounding factors must be considered when interpreting current human microbiota studies. One major limitation is inter-individual variability, which can arise from differences in ethnicity, genetic background, age, and environmental exposures. Ethnicity and host genomics have been shown to shape microbial composition and influence metabolite profiles, which may alter cancer susceptibility and progression. For instance, populations of African, Asian, or European descent harbor distinct microbial ecosystems that could modify PCa risk differently (94). African-American and Caucasian men with PCa showed enrichment of *Bacteroides* and *Streptococcus* accompanied by upregulated folate and arginine metabolism (95), whereas Japanese cohorts exhibited increased *Alistipes* and *Lachnospira*, taxa linked to interleukin-6-mediated inflammation and context-dependent SCFA production, highlighting ethnicity-specific microbial signatures that might differentially influence PCa risk (52).

Additionally, prior or ongoing antibiotic use—a common scenario in cancer patients—can profoundly disrupt microbial diversity and reduce the abundance of beneficial taxa, confounding associations between microbiota and treatment outcomes (96). Similarly, aging is associated with a progressive decline in microbial diversity and increased pro-inflammatory taxa, which may interact with PCa pathogenesis and modulate therapeutic responses.

Many human studies are observational or cross-sectional in design and involve small cohorts, thereby limiting causal inference. Furthermore, variability in sequencing methods, sample processing, dietary assessment, and lifestyle factors further compromises reproducibility. Therefore, future research should implement rigorous stratification, comprehensive metadata collection, and longitudinal sampling to better control for these confounders and strengthen the translational relevance of PCa–microbiota research.

## 8 Conclusion

The relationship between diet, gut microbiota, and PCa progression is of great importance and this review emphasizes how nutrition affect PCa outcomes. Dietary components like fats, red meat, and plant-based foods impact tumor growth. Inflammation and oxidative stress, influenced by diet, are also key factors in PCa outcomes. The gut microbiota exerts an important influence on PCa progression and treatment response, with dysbiosis linked to poor outcomes. Dietary interventions, such as prebiotics, probiotics, and personalized nutrition, show promise in modulating the microbiota to improve treatment effectiveness and prognosis of PCa. Clinicians should integrate diet and gut health into patient care, focusing on balanced nutrition to support a healthy microbiota and slow cancer progression.

Despite promising associations, the current evidence is predominantly derived from preclinical or non-PCa-specific research. Future investigations should aim to conduct metagenomic profiling of microbiota in PCa cohorts under active surveillance and design randomized controlled trials (RCTs) that stratify dietary interventions based on microbiota composition. Such approaches will enhance our understanding of personalized diet–microbiota strategies in PCa prevention and treatment.
